# Development of a Rapid Diagnostic Test to Distinguish between Emerging Viruses That Cause Hemorrhagic Fever

**DOI:** 10.4269/ajtmh.25-0168

**Published:** 2025-09-18

**Authors:** Andrew Wilson, Heather Poeck-Goux, Madison Ruschaupt, Scott Olschner, Keersten M. Ricks, Darci R. Smith

**Affiliations:** ^1^ Microbiology and Immunology Department, Biological Defense Research Directorate, Naval Medical Research Command, Fort Detrick, Maryland;; ^2^ Health and Biosciences, Parsons Corporation, Centreville, Virginia;; ^3^ Diagnostic Systems Division, U.S. Army Medical Research Institute for Infectious Diseases, Fort Detrick, Maryland

## Abstract

Viruses that cause the clinical syndrome referred to as viral hemorrhagic fever (VHF) are responsible for numerous infectious disease outbreaks. High-priority emerging viruses include orthoebolaviruses, orthomarburgviruses, Lassa virus, Crimean–Congo hemorrhagic fever virus, Rift Valley fever virus (RVFV), dengue virus (DENV), and yellow fever virus (YFV). Many of these viruses cause a similar clinical presentation in infected humans and have an overlapping geographic distribution with a risk of coemergence. As such, an antigen rapid diagnostic test to distinguish between these viruses would be beneficial in low-resource settings. In this study, we developed single-plex and multiplex antigen detection lateral flow immunoassays (LFIs) to rapidly detect and distinguish between emerging viruses that can cause VHF. We evaluated two antibody-labeling methods, colloidal gold nanoparticles and cellulose nanobeads (CNBs), to determine which approach would increase assay performance and multiplexing capabilities. Assay performance was evaluated by determining their sensitivity, specificity, matrix evaluation, and stability testing. All assays were highly specific, with no crossreactivity observed for the single-plex assays. Several of the assays performed better with the CNBs, including the DENV, YFV, RVFV, and orthomarburgvirus LFIs. No matrix effect was observed with most of the assays except that serum did impact the RVFV and DENV assays. In general, the multiplex assays were less sensitive compared with their respective single-plex assay. The most successful assays were the single-plex CNB LFIs assembled into an eight-plex cartridge, which allows for rapid and simultaneous testing of antigen to seven viruses.

## INTRODUCTION

Viruses associated with the clinical syndrome referred to as viral hemorrhagic fever (VHF) are responsible for a significant number of infectious disease outbreaks. Viral hemorrhagic fever is a syndrome of acute febrile illness characterized by increased vascular permeability that may lead to shock, coagulation defects, and bleeding.^1^ Important viruses that can cause VHF include dengue virus (DENV), yellow fever virus (YFV), Rift Valley fever virus (RVFV), Crimean–Congo hemorrhagic fever virus (CCHFV), Lassa virus (LASV), orthoebolaviruses (OEVs), and orthomarburgviruses (OMVs). Several of these viruses that can cause VHF share an overlapping geographical distribution, particularly in Africa, and there is a risk of coemergence.^2^

Dengue virus is transmitted by mosquitoes, and the clinical presentation ranges from asymptomatic to a mild febrile illness (dengue fever) to severe and potentially life-threatening diseases known as dengue hemorrhagic fever and dengue shock syndrome. At least 2.5 billion people worldwide are at risk for being infected with DENV. Severe disease from DENV infection is apparent in more than 100 countries, including those in the Americas, Asia, Africa, and Australia.^3^ Dengue virus exists as four antigenically similar but distinct serotypes (DENV1–4) that allow for productive infection with viruses from each of the four serotypes because of a lack of crossprotective antibodies.

Yellow fever virus is also transmitted by mosquitoes and can result in disease ranging from febrile illness to severe hemorrhagic disease and death. The distribution of YFV occurs in tropical regions of South America and Africa, where it is thought to cause around 109,000 severe infections per year with some 51,000 deaths.^4^ A safe and effective vaccine is available to prevent YFV infections, but outbreaks continue to occur because of a combination of factors that include inadequate vaccination coverage, weaknesses in public health infrastructure, and vaccine supply shortages.^5^

Rift Valley fever virus has caused severe epidemics and epizootics throughout Africa and the Arabian Peninsula.^6^ Severe outbreaks have involved tens of thousands of human and livestock cases. Human infections result from the bite of infected mosquitoes or contact with tissues, blood, or fluids from infected animals. Human disease is usually mild, and recovery occurs without major consequences. About 1–2% of infected individuals develop severe illness, with a case fatality ratio of 10–20% in hospitalized individuals.^7^

Crimean–Congo hemorrhagic fever virus is primarily maintained in ticks of the *Hyalomma* genus of the Ixodidae family, with a wide distribution spanning western, central, and southern Africa; the Balkans; the Middle East; southern Russia; and western Asia.^8^ Humans become infected by tick bites, through contact with a patient during the acute phase of infection, or through exposure to the blood or tissues of viremic livestock.^9^^,^^10^ The case fatality rate is approximately 30% during the hemorrhagic phase of the disease, but this can vary by geographical location.^11^

Lassa virus can cause Lassa fever and is endemic in Nigeria, Sierra Leone, Guinea, and Liberia; additionally, sporadic cases occur in other West African countries. Infection occurs because of exposure to excreta and other direct contact with the multimammate rat (*Mastomys natalensis*). Mortality in these hyperendemic regions is estimated to be as high as 300,000 infections and 10,000 deaths annually, with 15–20% of hospitalized patients succumbing to the disease.^12^

Orthoebolaviruses and OMVs are filoviruses that can cause Ebola virus (EBOV) disease and Marburg virus disease (MVD), respectively. These viruses are highly pathogenic and have been associated with devastating outbreaks causing case fatality ranging from 25% to 90%.^13^ Bats are most likely the reservoir of filoviruses, and the geographical distribution of both viruses appears to be limited primarily to sub-Saharan Africa.^14^^,^^15^ Outbreaks are usually the result of person-to-person transmission that occurs through direct contact with blood and other body fluids.

Collectively, these viruses that can cause VHF share similarities in early clinical manifestations with other febrile illnesses, such as generalized malaise and fever, which are similar to other common tropical diseases. The laboratory diagnosis of acute VHF usually occurs early in the symptomatic phase during the first 3–4 days. Viremia can be brief, which presents a challenge for viral antigen detection assays. Reverse transcription polymerase chain reaction (RT-PCR) is commonly used for identifying the viruses that cause VHF, but these emerging viruses occur in low-resource settings that sometimes lack advanced laboratory infrastructure. The lateral flow immunoassay (LFI) provides an attractive option as a rapid antigen detection assay that can be used in these low-resource settings because it is easily deployable, is user friendly, does not require electricity, and is shelf stable. As was observed during the coronavirus disease 2019 pandemic, LFIs have the potential to help change the trajectory of epidemics and pandemics because diagnostic results can be delivered to the point of need.^16^ Furthermore, these antigen assays are less susceptible to viral drift or evolution because accumulated mismatches in the primers and probes used in RT-PCR can impact the performance of molecular diagnostic assays. Various individual LFIs have been developed to detect viruses that cause VHF with varying degrees of success.^17^^–^^29^ However, these assays have never been combined into a multiplex LFI platform. An LFI panel that can simultaneously detect and distinguish between DENV, YFV, RVFV, CCHFV, LASV, OEVs, and OMVs would be highly advantageous for surveillance activities in remote settings.

Colloidal gold nanoparticles (AuNPs) are one of the most commonly used labels in lateral flow because of their ease of use and the availability of good commercial sources. That being said, a major limitation of AuNP is the lack of color choices, which makes multiplexing more challenging. New labeling technology, such as cellulose nanobeads (CNBs), is available in several colors for multiplexing applications. Furthermore, the use of CNBs has shown an increase in sensitivity when compared with the use of AuNP as the label.^30^ In this study, we evaluated two antibody-labeling methods to determine which approach would increase assay performance and multiplexing capabilities. Collectively, we developed sensitive and specific LFIs to rapidly detect seven viruses that share an overlapping geographic distribution throughout Africa. This is the first multiplex LFI panel developed for the differential diagnosis of VHF.

## MATERIALS AND METHODS

### Lateral flow immunoassay test strip configuration.

**Figure 1.**
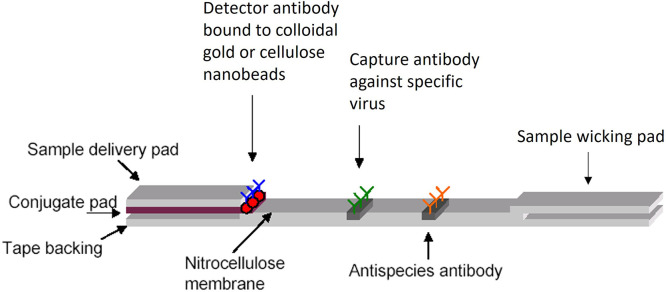
Basic components of the lateral flow immunoassay.

The LFI test strip configuration (Figure 1) consists of overlapping membranes that are mounted on a backing card for stability. The liquid sample is applied at one end of the strip on the adsorbent sample pad. The sample migrates through the conjugate release pad that contains virus-specific antibodies, which are conjugated to AuNP (BBI Solutions gold conjugation services, Crumlin, United Kingdom) or CNB (DCN Diagnostics, Carlsbad, CA) following the manufacturers’ recommendations. The AuNP-labeled detector antibody is lyophilized on a polyester strip treated with bovine serum albumin (BSA) and sodium chloride. The CNB-labeled detector antibody is oven dried on a polyester strip treated with BSA and sodium chloride. The sample along with the conjugated antibody that is bound to the target analyte then migrates along the strip to the detection zone. This membrane is composed of nitrocellulose with target-specific antibodies immobilized in lines where the capture antibodies are sprayed on the membrane using a BioDot XYZ3060 (BioDot, Irvine, CA). The nitrocellulose membrane with applied antibodies is blocked using a polyvinyl alcohol solution to minimize nonspecific interactions. These antibodies react with the viral antigen bound to the conjugated antibody. A colorimetric response on the test line results when the viral antigen is detected, whereas a response on the control line indicates the proper liquid flow through the strip. The assay is designed to produce a visible result in 15 minutes, and a result is considered positive when both a control line and a test line are visible.

### Antibody pairs.

The antibody pairs used to develop the individual antigen detection LFIs were either commercially available or developed internally at U.S. Army Medical Research Institute for Infectious Diseases (USAMRIID), Naval Medical Research Command (NMRC), or the Centers for Disease Control and Prevention (Table 1). Optimal antibody pairs were selected based on screening various combinations by ELISA or spot testing on LFI strips. The DENV assay used commercially available antibodies from Abcam (Cambridge, United Kingdom) and ATCC (Manassas, VA). The YFV assay used commercially available antibodies from the Native Antigen Company (Oxford, United Kingdom). The AuNP-based RVFV assay used a polyclonal and monoclonal antibody developed by NMRC. The CNB-based RVFV assay used a monoclonal antibody developed by USAMRIID. The CCHFV, LASV, and OMVs assays used monoclonal antibodies developed by USAMRIID. The OEVs assay used the previously published antibody pair.^19^

**Table 1:** The antibody pairs and viral protein targets for the colloidal gold nanoparticle and cellulose nanobead lateral flow immunoassays

Virus (abbreviation)	Antibodies (source)	Viral Protein Target
DENV-1	Capture: ab9202 (Abcam) and 1-H10-6 (ATCC HB-48); detector: D1-4G2-4–15 (ATCC HB-112)	NS1
DENV-2		
DENV-3		
DENV-4		
YFV	Capture: HH7 (native antigen); detector: CE6 (native antigen)	NS1
RVFV	AuNP format: capture: rabbit anti-RVFV poly (NMRC); detector: RVF-01-A-2A (NMRC) CNB assay format: capture: R3624 P2G12 (USAMRIID); detector: R3624 P2G12 (USAMRIID)	Polyclonal/NP (AuNP format) and NP (CNB format)
CCHFV	Capture: 12G10-2-2 (USAMRIID); detector: 5G2-1-1A (USAMRIID)	NP
LASV	Capture: L52-2159-15 (USAMRIID); detector: L-52–189-5 (USAMRIID)	NP
OMVs	Capture: R4640-3MI1 (USAMRIID); detector: R4668-X-FB05-BH11 (USAMRIID)	VP40
OEVs	Capture: rabbit anti-Ebola poly (NMRC); detector: B-MD04-BD07_AE11 (CDC)	Polyclonal/VP40

AuNP = colloidal gold nanoparticle; ATCC = American Type Culture Collection; CCHFV = Crimean–Congo hemorrhagic fever virus; CNB = cellulose nanobead; DENV = dengue virus; LASV = Lassa virus; NMRC = Naval Medical Research Command; NP = nucleoprotein; OEV = orthoebolavirus; OMV = orthomarburgvirus; RVFV = Rift Valley fever virus; USAMRIID = U.S. Army Medical Research Institute for Infectious Diseases; YFV = yellow fever virus.

### Single-plex and eight-plex cartridges.

The single-plex cartridges were acquired from DCN Diagnostics and were designed to contain a 5-mm test strip. The eight-plex cartridge was custom made by HDT Global (Fredericksburg, VA) to contain the same size: a 5-mm test strip. A sonic welder (Sonics & Materials Inc., Newtown, CT) was used to secure the strips in this configuration using custom parameters set by the manufacturer.

### Lateral flow immunoassay testing.

The performance of the LFIs was evaluated using recombinant protein, inactivated virus stocks, and infectious virus stocks. Multiple concentrations were evaluated in duplicate to define the assay range and estimated limit of detection (LOD). The typical range tested for virus stocks was 5- and 10-fold dilutions and 31.3–2.5 ng/mL for recombinant protein. Samples were diluted in a running buffer containing phosphate-buffered saline (PBS) and 0.1% triton X-100, and 100 *µ*L were added per strip. The LOD for each LFI is defined as the lowest level producing a positive result for both replicates. For specificity testing, an inclusivity/exclusivity panel was curated that included multiple representative virus strains and near neighbors that were tested in duplicate.

#### Recombinant proteins.

Recombinant LASV and CCHFV nucleoproteins (NPs) were from USAMRIID. Musoke–Marburg virus-like particles (VLPs) were obtained commercially (Creative Diagnostics, Shirley, NY). Dengue virus VLP serotypes 1–4, YFV NS1 recombinant antigen, and RVFV NPs were purchased from The Native Antigen Company (Kidlington, United Kingdom).

#### Inactivated virus stocks.

Inactivated virus stocks, produced by USAMRIID, were used to determine inclusivity/exclusivity performance of the LFIs before testing in the BSL3/4 suites. Viruses were inactivated using a combination of chemical (0.3% beta-propiolactone) and gamma irradiation (minimum dose of 2 million rads from a cobalt irradiator). The list of inactivated viruses used is detailed in Supplemental Table 1. For most stocks, the strain and titer (plaque-forming units [PFUs] per milliliter) were known. Only a few historical stocks did not have titers or specific strain information.

#### Infectious virus stocks.

Virus stocks for DENV were provided by Daniel Ewing from NMRC and included DENV-1 Western Pacific 74, DENV-2 OBS8041, DENV-3 CH53489, and DENV-4 H241 isolates. Infectious DENV was handled under biosafety level 2 conditions. The YFV was obtained through BEI Resources, National Institute of Allergy and Infectious Diseases (NIAID), NIH as part of the World Reference Center for Emerging Viruses and Arboviruses program: YFV, CAREC M2-09, and NR-50062. The RVFV was obtained through BEI Resources, NIAID, NIH: RVFV, ZH501, and NR-37378. A virus stock was created for YFV and RVFV by one passage of the virus obtained from BEI Resources on Vero-81 cells. Infectious YFV and RVFV were handled under BSL-3 conditions. Infectious BSL-4 viruses were provided from USAMRIID and included the following strains: OMV (Musoke, Angola, Ravn, and Ci67), LASV (Macenta, Pinneo, Josiah, and Weller), CCHFV (Ibr101200, Afg09, DAK8193, UG3010, Drosdov, SPU 128/81/7, PAK JD206, Chinese HY-12, and Hoti), and OEV (Mayinga, Bundibugyo, Reston, Gulu, and Boniface). Infectious OMVs, OEVs, LASV, and CCHFV were handled under BSL-4 conditions.

#### Matrix evaluation (interfering substances).

Several different matrices were evaluated to assess potential assay interference. We evaluated human serum and matrices associated with environmental sampling (soil, tap water, sewage, dust, and mosquito homogenate for the arbovirus assays). All matrices were prepared in the LFI test running buffer with the exception of tap water. Pooled normal human serum (NRL BioSpec, Melbourne, Australia) was diluted 1:5, sewage (collected from a local wastewater treatment plant) and dust (collected from our building) were diluted 1:1,000 from a 1-mg/mL stock vial, and soil (Princeton soil-2003–110 North American Proficiency Testing, Madison, WI) was prepared by mixing 1 g of soil with 6 mL of buffer and allowed to settle for 15 minutes before removing the top liquid portion for testing. Tap water was collected directly from our building’s faucet. The mosquito homogenate was prepared by homogenizing 25 *Aedes aegypti* mosquitoes in 1 mL of PBS and centrifuging to remove tissue debris, and the supernatant was removed for testing. Each matrix was evaluated at one time the LOD with and without virus in duplicate. Recombinant protein and/or inactivated virus stocks were used for matrices testing.

#### Stability testing.

The stability of the assays was evaluated by artificially accelerating exposure to increased temperatures to mimic possible temperature changes in the field. The individual assays were stored in heat-sealable aluminum bags with desiccants and placed in an incubator at 45°C for various lengths of time. For 8 weeks, six assay strips per week were evaluated at two times and one time the LOD in buffer.

## RESULTS

### Assay development and performance.

We developed individual antigen detection LFIs for all four serotypes of DENV, YFV, RVFV, CCHFV, LASV, OMVs, and OEVs using antibody pairs that were either commercially available or developed internally at NMRC or USAMRIID. We maintained the previously published OEV LFI for inclusion in this project.^19^ Assays were developed in two formats, where the detector antibody was bound to either AuNPs or CNBs.

The performances of the seven individual LFIs were evaluated using an inclusivity/exclusivity panel of inactivated virus supernatants (Supplemental Table 1). All assays were highly specific, with no crossreactivity observed. The DENV LFI detected all four serotypes, whereas the OEV and OMV assays detected multiple isolates. The OEV LFI was previously shown to detect EBOV, Sudan virus, Taï Forest virus, and Reston virus.^19^ We have further demonstrated that the EBOV LFI detects Bundibugyo virus, and we observed a similar LOD as previously reported. The LASV LFI detected all isolates except for Pinneo.

**Figure 2.**
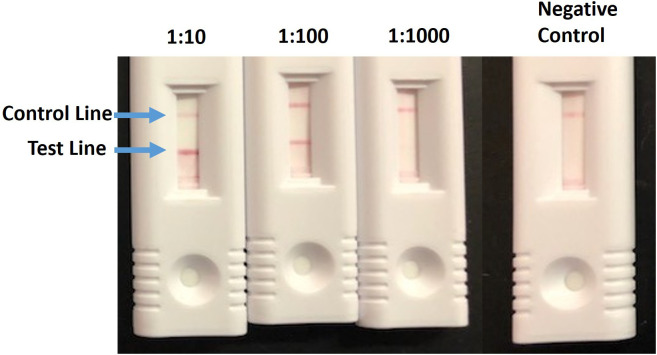
The orthomarburgvirus lateral flow immunoassay tested at multiple concentrations using inactivated virus stock material.

The LOD of the seven individual AuNP LFIs was determined using recombinant protein, inactivated virus supernatants, or infectious virus supernatants (Table 2). The DENV LFI ranged in sensitivity for the different serotypes, with DENV-2 and DENV-4 being most sensitive when testing recombinant protein (15.6–31.3 ng/mL) and infectious virus (4.3–6.1 log_10_ PFU/mL). The YFV and RVFV LFIs were less sensitive compared with the other assays, whereas the LASV and Marburg virus (MARV) assays were the most sensitive. The YFV AuNP LFI detected 6.3 log_10_ PFU/mL of infectious virus. The RVFV LFI was only able to detect 6.6 log_10_ PFU/mL of inactivated virus and was unable to detect infectious virus. In contrast, the LASV and OMV LFIs were substantially more sensitive and were able to detect 2.7 and 4.6 log_10_ PFU/mL of infectious virus, respectively. An example of the OMV LFI is shown in Figure 2 and is representative of the appearance of the control and test lines for the other assays.

**Table 2:** The limit of detection of the individual colloidal gold nanoparticle lateral flow immunoassays to seven viruses

Virus	LOD (recombinant protein; ng/mL)	LOD (inactivated virus; log_10_ PFU/mL)	LOD (infectious virus; log_10_ PFU/mL)
DENV-1	31.3	4.9	6.0
DENV-2	15.6	6.0	5.0
DENV-3	31.3	4.3	6.1
DENV-4	15.6	5.4	4.3
YFV	Not tested	6.1	6.3
RVFV	Not tested	6.6	Not detected
CCHFV (Afg09)	2.5	5.7	6.2
LASV (Josiah)	3.9	4.3	2.7
RAVV (Ravn)	28.5	4.3	4.6
EBOV	Not tested	6.5	4.7

CCHFV = Crimean–Congo hemorrhagic fever virus; DENV = dengue virus; EBOV = Ebola virus; LASV = Lassa virus; LOD = limit of detection; PFU = plaque-forming unit; RAVV = Ravn virus; RVFV = Rift Valley fever virus; YFV = yellow fever virus.

We evaluated several different matrices to assess potential assay interference. Human sera and matrices associated with environmental sampling (soil, tap water, sewage, dust, and mosquito/tick homogenate for the arbovirus assays) were evaluated. No matrix effect was observed with most of the assays, and the expected detection limit was successfully achieved. However, serum did impact the RVFV and DENV assays by a factor of two times the LOD (Supplemental Table 3). All assays were evaluated for stability by artificially accelerating exposure to increased temperatures to mimic possible temperature changes in the field. All seven assays were found to maintain stability, with no decrease in the expected level of sensitivity.

**Figure 3.**
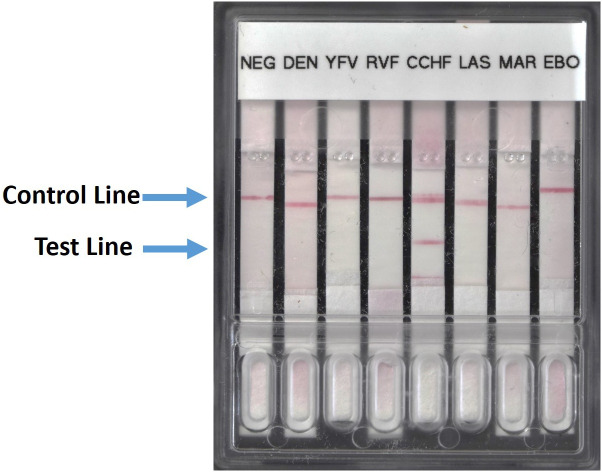
Individual colloidal gold nanoparticle lateral flow immunoassays assembled into an eight-plex cartridge where the Crimean–Congo hemorrhagic fever virus (labeled CCHF) strip tested positive. DEN = dengue virus; EBO = Ebola virus; LAS = Lassa virus; MAR = Marburg virus; NEG = negative; RVF = Rift Valley fever virus; YFV = yellow fever virus.

Next, we assembled the seven individual assays and a negative control strip in an eight-plex cartridge to allow for greater simultaneous testing capabilities. The individual assay strips were arranged within the custom-made cartridge and welded together. We confirmed the performance characteristics in this format, and all assays performed the same in the eight-plex cartridge compared with the individual assays in single-plex cartridges. The LODs remained the same as described in Table 2 when the individual assays were assembled in the eight-plex cartridge. The advantage of this configuration is that it allows for easier testing of unknown samples for the seven viruses of interest. The same sample can quickly be added to the cartridge to allow for the simultaneous testing of the virus antigen. A representative image of the eight-plex cartridge is depicted in Figure 3.

### Evaluate new detector antibody-labeling technology and multiplex capabilities.

**Figure 4.**
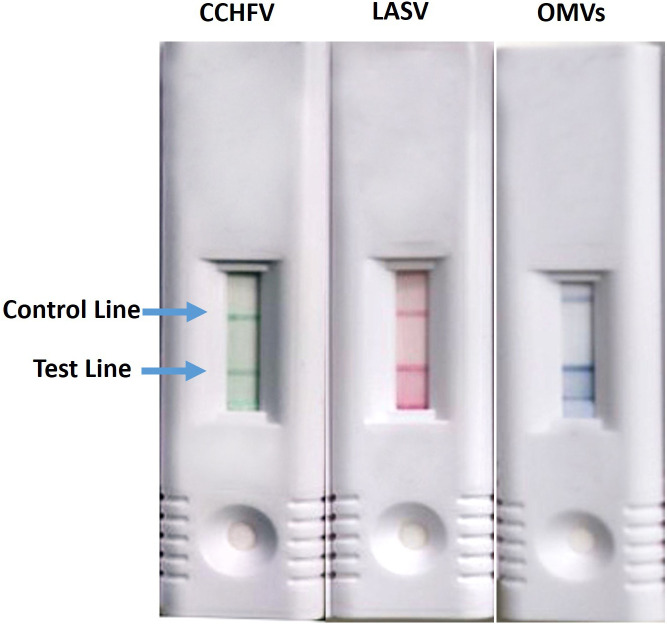
Individual cellulose nanobead assays were developed using green, red, or blue beads. CCHFV = Crimean–Congo hemorrhagic fever virus; LASV = Lassa virus; OMV = orthomarburgvirus.

Next, we evaluated new antibody-labeling technology in hopes of increasing the sensitivity of the assays and to provide greater multiplexing capabilities. We optimized the use of the CNBs, which came in red, blue, or green beads. First, we developed individual assays for all seven viruses. An example of the CCHFV, LASV, and OMVs LFIs and is shown in Figure 4 is representative of what the control and test lines look like for each color of CNBs used. The CNB single-plex assays were either comparable or more sensitive when compared with the AuNP assays (Table 3). The new CNB single-plex assays had comparable sensitivities to the AuNP LFIs when evaluating the LOD with recombinant protein. In contrast, several of the CNB assays performed better than the AuNP assays when evaluating the LOD with inactivated or infectious virus. The assays that performed better include the DENV LFI to all four serotypes and the YFV, RVFV, CCHFV, and OMVs LFIs. The DENV LFI increased in sensitivity an average of 1.2 log_10_ PFU/mL depending on the serotype tested when testing infectious virus. The YFV, CCHFV, and OMVs LFIs demonstrated increases in sensitivity by 1.0, 0.7, and 1.3 log_10_ PFU/mL, respectively, with the use of the CNBs when testing infectious virus. The same antibody pair used in the AuNP RVFV LFI was not successful with the CNBs, so a new capture–detector pair using USAMRIID-provided antibodies was identified with ELISA checkerboard experiments and found to be sensitive.

**Table 3:** The limit of detection of the individual and multiplex cellulose nanobead lateral flow immunoassays to seven viral hemorrhagic fevers

Virus	Single-plex LOD (recombinant protein; ng/mL)	Single-plex LOD (inactivated virus; log_10_ PFU/mL)	Single-plex LOD (infectious virus; log_10_ PFU/mL)	Multiplex LOD (recombinant protein; ng/mL)	Multiplex LOD (inactivated virus; log_10_ PFU/mL)	Multiplex LOD (infectious virus; log_10_ PFU/mL)
DENV-1	31.3	3.9	4.7	31.3	3.9*	4.7
DENV-2	15.6	5.0	3.8	15.6	5.0*	4.1
DENV-3	31.3	3.3	5.1	31.3	3.3*	5.1
DENV-4	15.6	4.4	3.1	15.6	4.4*	3.4
YFV	Not available	6.1	5.3	Not available	6.1*	5.3
RVFV	250	5.8	3.3	500	5.8*	4.0*
CCHFV (Afg09)	1.25	5.7	5.5	2.5	5.7	6.8
LASV (Josiah)	3.9	4.3	3.4	8	4.3	3.4
OMV (Ravn)	28.5	2.6	3.3	28.5	3.3	4.8
OEV (Mayinga)	Not available	6.5	5.5	Not applicable	Not applicable	Not applicable

CCHFV = Crimean–Congo hemorrhagic fever virus; DENV = dengue virus; LASV = Lassa virus; LOD = limit of detection; OEV = orthoebolavirus; OMV = orthomarburgvirus; PFU = plaque-forming unit; RVFV = Rift Valley fever virus; YFV = yellow fever virus.

* Crossreactivity was observed to the other viruses on the strip.

We expanded our testing of the AuNP and CNB LFIs for CCHFV, LASV, and OMVs to evaluate additional isolates. Multiple isolates were detected for all three LFIs except that the LASV LFI was not able to detect the Pinneo isolate (Supplemental Table 2). The CNB LFIs continued to typically show an increase in sensitivity when compared with the AuNP LFI format. Collectively, these results demonstrate that the CNB LFI format typically increases the performance of the assays, especially when testing infectious virus.

**Figure 5.**
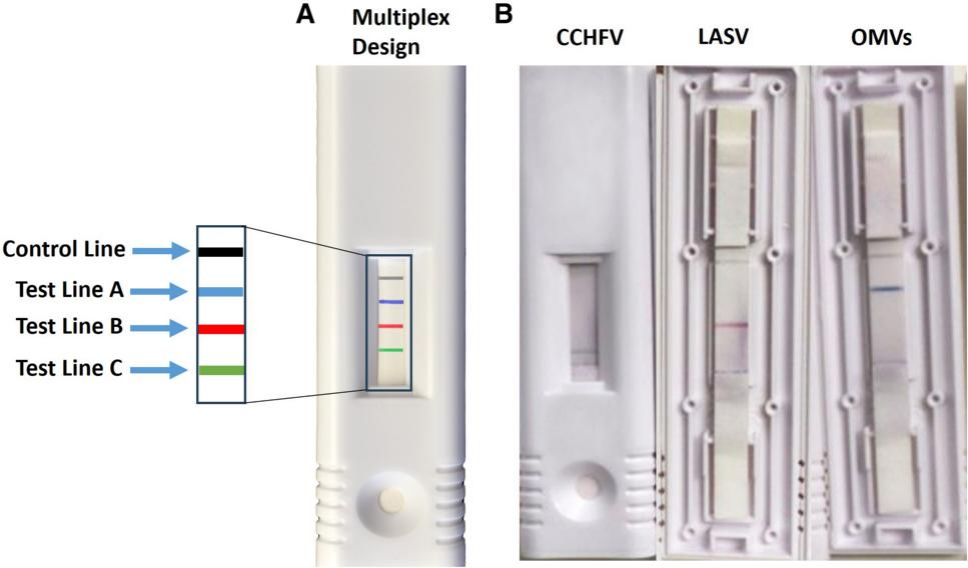
Multiplex colloidal gold nanoparticle lateral flow immunoassays were developed using green, red, or blue beads. (**A**) Multiplex strip design for three targets. (**B**) An example of the multiplex lateral flow immunoassay testing positive for Crimean–Congo hemorrhagic fever virus (CCHFV), Lassa virus (LASV), or orthomarburgviruses (OMVs). The cartridge was opened for two of the test strips to better show visibility of the control line.

Because the use of the CNB LFI format proved successful, we explored the ability to multiplex the assays onto one strip. The size of the LFI cassette allowed for three test lines and one control line per strip, so we developed one strip that would detect OMVs, LASV, or CCHFV and another strip that would detect DENV, YFV, or RVFV (Figure 5A). We left the OEV LFI as an individual assay because it has already undergone extensive testing with human samples.^19^ Additionally, there are numerous other single-plex LFIs available for OEVs and fewer for the other viruses in this panel. However, it would still be beneficial to include an OEV LFI in a multiplex format in future panels that are developed. Representative images of the multiplex assays testing positive for each virus are shown in Figure 5B, and they are representative of what the control and test lines look like for each color of CNB used. It was often difficult to see the control line in the cartridge window for the multiplex format, so the cartridge was opened to more clearly show the lines. We determined the LOD of the multiplex assays with recombinant protein, inactivated virus, and infectious virus (Table 3). In general, the multiplex assays were less sensitive compared with the single-plex assays. When comparing LODs with infectious virus between single-plex and multiplex LFIs, the CCHFV and OMV multiplex LFIs performed the worst and detected 1.3 and 1.5 log_10_ PFU/mL less virus, respectively. We also observed some crossreactivity with the DENV, YFV, and RVFV multiplex strip. Interestingly, the crossreactivity was inconsistent when analyzing recombinant protein versus inactivated or infectious virus. The crossreactivity is likely because of the interference of the different antibodies as they migrate on the same strip.

## DISCUSSION

Rapid diagnostic tests (RDTs) are urgently needed for high-priority pathogens with epidemic emergence potential, such as the viruses that can cause VHF. Individuals infected with these viruses usually present with similar signs and symptoms, making their diagnosis problematic. Distinguishing viruses that can cause VHF from other tropical diseases is important for isolation, infection control procedures, and disease management. Here, we describe the first sensitive and specific LFI panel to rapidly detect and distinguish between seven viruses that can cause VHF with overlapping geographical distribution throughout Africa.

Differential diagnosis of VHF can be problematic, particularly in low-resource settings. Currently, there are limited commercially available molecular diagnostics that can detect multiple pathogens that cause febrile illness.^31^ No multiplex rapid diagnostic assays currently exist to detect multiple viruses that cause hemorrhagic fever. Lateral flow immunoassays are widely used for RDTs, but all of the existing assays are limited to being able to detect a single virus.

There have been a number of RDTs developed for DENV that are commercially available and various studies that have compared their performance (reviewed in ref. 28). Similar to the design of our assay, the commercially available RDTs detect NS1, and many of them are combined with a rapid assay to detect antibodies concurrently with viral antigen. Differing sensitivities have been reported based on the infecting DENV serotype for some commercial assays, where most had a lower sensitivity for DENV-4.^32^ This is in contrast to our results, where our DENV assay was most sensitive to detect DENV-2 and DENV-4. It is likely that the different antibodies used in the various assays bind to the different serotypes with varying affinities.

Few reports have described the development of a diagnostic test for YFV based on the detection of NS1 antigen.^33^ No commercially available RDTs are available to detect YFV antigen, and only laboratory-developed LFIs have been described thus far. Yen et al.^34^ developed a multiplexed silver nanoparticle-based LFI that included DENV and YFV NS1 detection. The assay was able to detect 150 ng/mL of viral protein but was not evaluated with infectious virus or clinical samples. Kim et al.^27^ developed an RDT that was tested with positive and negative samples of monkeys from Brazil, and the assay was able to detect 1.6 PFU/mL of infectious virus from cell culture supernatant. Our YFV single-plex CNB LFI had an LOD of 5.3 log_10_ PFU/mL, which is not as sensitive as some of our other assays developed as part of this project, but we were limited to the use of commercially available antibodies. The development of better-affinity reagents for YFV would be beneficial.

For RVFV, an LFI has been developed to detect infected mosquitoes.^22^ Additionally, one commercially available RDT exists for RVFV for testing serum or whole blood from cattle and small ruminants. The ID Rapid^®^ Rift Valley Fever Antigen test is produced by Innovative Diagnostics (Montpellier, France) and is based on the rapid pen-side test described by Cêtre-Sossah et al.^25^ This LFI was developed to detect the NP of RVFV, and the LOD ranged from 4.2 to 6 log_10_ PFU/mL depending on the virus strain tested.^25^ In comparison, our single-plex CNB LFI had an LOD of 3.3 log_10_ PFU/mL, suggesting that it may be more sensitive.

Currently, only one commercially available RDT exists for CCHFV to detect IgM-specific antibodies in the patient’s plasma.^35^ No commercially available RDTs exist to detect CCHFV antigen. A laboratory-designed assay was described using a high-affinity nonantibody binding protein called affimer, which was developed to recognize the NP of CCHFV.^17^ Their reported LODs spiking horse sera with different concentrations of recombinant NP were 15 ng/strip or 750 ng/mL. Our single-plex CNB LFI had an LOD of 1.25 ng/mL, which is substantially more sensitive. Thompson et al.^29^ described an antigen RDT that was evaluated prospectively on serum samples from patients with suspected CCHFV infection in Türkiye and retrospectively on stored serum samples in Iraq. The LOD was not described, but the assay was found to be sensitive and specific when compared with reverse transcription quantitative polymerase chain reaction.^29^

Lassa virus has multiple lineages, which can make designing diagnostic assays challenging because of its high genetic diversity. For LASV, there is currently one commercially available RDT kit, the ReLASV *Pan*-Lassa Antigen Rapid Test (Zalgen Labs, Frederick, MD), which detects the LASV NP from lineages II, III, and IV.^24^ The ReLASV Antigen Rapid Test^23^ was discontinued, likely because it only detected the Josiah isolate. The ReLASV *Pan*-Lassa Antigen Rapid Test (Zalgen Labs) is currently research use only, and to the best of our knowledge, the LODs have not been described. However, among samples that were tested during a Lassa fever outbreak in Nigeria, 100% of samples that gave a cycle threshold value below 22 by two real-time RT-PCR assays were positive using the ReLASV *Pan*-Lassa Antigen Rapid Test (Zalgen Labs).^24^ Our LASV LFI also did not detect a lineage I strain (LASV Pinneo) but did detect lineages III and IV; however, a lineage II strain was not tested. Our results demonstrate how the sequence divergence of the various LASV lineages can make it difficult to find conserved regions suitable for antibody-based detection.

Several LFIs have been developed for OEVs in part because of the 2014–2016 EBOV outbreak, which led to six RDTs being commercially available and many other laboratory-designed tests.^36^^,^^37^ The OEV LFI used in this study was tested with clinical samples during an outbreak in Liberia and had an 87.9% sensitivity and 96.8% specificity for both plasma and oral swab samples when using real-time RT-PCR as the reference test.^19^ The LOD was 4.7–5.7 log_10_ PFU/mL, similar to what we have described in this study. The anti-EBOV polyclonal antibody used in this laboratory-designed assay was licensed to OraSure (Bethlehem, PA) for commercial development. OraSure developed the OraQuick Ebola Rapid Antigen Test that was approved by the U.S. Food & Drug Administration. The LOD in whole blood for inactivated EBOV was determined to be 6.2 50% tissue culture infectious dose (TCID_50_)/mL.^38^

There is currently one commercially available RDT for MVD called NOVA test by Atlas Link Technology (Beijing, China), but the assay performance data and regulatory status are not disclosed or available in the literature to the best of our knowledge. A laboratory-designed LFI was recently described and was able to detect 4–6 log_10_ TCID_50_/mL of MARV depending on the isolate and 5 log_10_ TCID_50_/mL of RAVV.^26^ We were able to detect 3.5–4 log_10_ PFU/mL of MARV depending on the isolate and 3.3 log_10_ PFU/mL of Ravn virus using our CNB LFI, which proved to be more sensitive compared with the AuNP LFI. That being said, it is difficult to directly compare titers determined by TCID_50_ assay versus a plaque assay. Regardless, the assays described by Changula et al.^26^ and our study highlight promising tests that could be further developed for the rapid diagnosis of MVD.

Collectively, the LFIs described in our study were more sensitive compared with those previously published, except for the YFV LFI (Table 4). Several of the assays performed better with the CNBs, including the DENV (DENV1–4), YFV, RVFV, and OMV LFIs. Cellulose nanobeads are often more sensitive than AuNPs for LFIs because of several factors related to their physical and chemical properties. Cellulose nanobeads have larger surface areas compared with AuNPs, which allow for a higher loading capacity of detection antibodies. This can increase assay sensitivity because more binding events can occur on the surface of each bead. The porous structure of the CNBs provides numerous binding sites, enhancing the capture and retention of target molecules. The CNBs come in multiple colors, so we were hopeful that this would increase our ability to multiplex the assay; however, we found it difficult to distinguish the colors when used on the same test strip. Additionally, some colors were easier to see than others, with blue being the most distinctive compared with the red and green particles. An important point for consideration when using these multiple colors for assay development is that an individual who is color blind would not be able to distinguish between them.

**Table 4:** Comparison of the limits of detection described in our study using the single-plex cellulose nanobead lateral flow immunoassays and infectious virus compared with those previously published

Target	Current Study LOD (log_10_ PFU/mL)	Previously Published LOD (log_10_)	References
DENV	3.1–5.1	Not reported*	28
YFV	5.3	0.2 PFU/mL	27
RVFV	3.3	4.2–6 PFU/mL	25
CCHFV	4.5–6.3	Not reported*	17 and 29
LASV	3.4–5.5	Not reported*	23 and 24
OEV	5.5	4.7–5.7 PFU/mL	19
OMV	3.3–4	4–6 TCID_50_/mL	26

CCHFV = Crimean–Congo hemorrhagic fever virus; DENV = dengue virus; LASV = Lassa virus; LOD = limit of detection; OEV = orthoebolavirus; OMV = orthomarburgvirus; PFU = plaque-forming unit; RVFV = Rift Valley fever virus; TCID = tissue culture infectious dose; YFV = yellow fever virus.

* Not reported with infectious virus.

Environmental sampling did not appear to impact the performance of the assays developed here, but serum did have a slight impact on the RVFV and DENV LFIs. Serum can impact immunoassays because of its complex composition, such as high protein content and endogenous antibodies that may interfere with the interaction between the specific antibodies used and the target antigen. All assays were highly stable when subjected to artificial acceleration of increased temperatures to mimic tropical conditions, suggesting that they will maintain their performance in the field when temperatures might fluctuate higher than room temperature. The use of RDTs is primarily used to identify infected individuals, but other applications include testing potential animal reservoirs and/or arthropods for the presence of the virus. During the 2014–2016 EBOV outbreak, the rapid screening of cadavers to support safe burial practices was crucial in controlling the outbreak and preventing further spread of the virus. These RDTs would be helpful in settings where nosocomial infections pose a risk to health care workers because bodily fluids could be rapidly tested.

One of the known limitations of LFI sensitivity for viral antigen detection is the high detection threshold where the LOD is generally in the low nanograms per milliliter range, which is what we report for the assays described here. We attempted several approaches to increase the sensitivity of the assays while still maintaining a form factor that can easily be used in field-based testing. Fluorescent-based LFIs are generally more sensitive compared with colorimetric LFIs, such as those developed here, but they require a reader that complicates field use and is generally more expensive. Screening of additional antibody pairs may also increase the performance of the assays. We did observe differences in detection sensitivity between inactivated and infectious virus stocks, which highlight the need to include both for comprehensive evaluation of LFI detection limits. We have observed this difference when testing other immunoassay formats, and this is likely because of changes in the epitope or antigen alteration during the inactivation process.

This study has several limitations. This work was primarily a development exercise, and additional testing with human samples, including clinical validation, is required. Furthermore, use of these LFIs as currently configured would require centrifugation of blood samples, which can be difficult in low-resource settings, although the assays can be configured to include a mechanism to remove red blood cells.^39^ Finally, LFIs, including the ones developed in this study, are generally less sensitive compared with RT-PCR assays. Lateral flow immunoassays generally require higher antigen concentrations (approximately nanograms per milliliter) than polymerase chain reaction needs for nucleic acids (femtograms to picograms range). Additionally, viral antigen levels may be below the detection limit in early stages before peak viremia or in late stages after immune clearance begins. This requires that the user understands the characteristics and limitations of the assay. For example, a positive test result would provide rapid information for patient isolation and disease management. In contrast, a negative test result would still require confirmatory analysis with a real-time RT-PCR assay, especially in the event of the presence of clinical signs and symptoms.

## CONCLUSION

In conclusion, we developed the first multiplex LFI panel for the differential diagnosis of VHF. A multiplex approach is a more useful and efficient strategy for surveillance, differential diagnosis, and outbreak determination rather than relying on assays specific for only one virus. These assays will be useful as rapid point-of-need tests followed by ELISA and real-time RT-PCR as confirmatory assays.

## Supplemental Materials

10.4269/ajtmh.25-0168 Supplemental Materials
