# Supplementary material for: Development of a Rapid Diagnostic Test to Distinguish between Emerging Viruses That Cause Hemorrhagic Fever
Source: Am J Trop Med Hyg. 2025 Sep 18;113(5):1097–105. doi: 10.4269/ajtmh.25-0168 (PMC12590983; doi:10.4269/ajtmh.25-0168)
Supplement: Supplemental Materials [file tpmd250168.SD1.pdf]

**Supplemental Table 1.** Inclusivity/exclusivity testing of the AuNP LFIs using inactivated virus stocks. All supernatants were diluted 1:2 in the LFI running buffer. Cells with a check mark indicate a positive LFI result and no check mark indicates a negative result.

| Virus | Isolate | Infectious Titer ($\log_{10}$ PFU/mL) | DENV LFI | YFV LFI | RVFV LFI | CCHFV LFI | LASV LFI | OEV LFI | OMV LFI |
|---|---|---|---|---|---|---|---|---|---|
| DENV 1 | WP74 | 5.9 | ✓ | | | | | | |
| DENV 2 | 21603 | 7.0 | ✓ | | | | | | |
| DENV 3 | CH55489 | 5.3 | ✓ | | | | | | |
| DENV 4 | 341750 | 6.4 | ✓ | | | | | | |
| YFV | ASIBI | 7.4 | | ✓ | | | | | |
| RVFV | ZH501 | 7.8 | | | ✓ | | | | |
| CCHFV | 10200 | 7.7 | | | | ✓ | | | |
| LASV | Macenta | 8.1 | | | | | ✓ | | |
| LASV | Josiah | 6.3 | | | | | ✓ | | |
| LASV | Pinneo | 7.3 | | | | | | | |
| EBOV | Mayinga | 7.5 | | | | | | ✓ | |
| RESTV | Reston | 6.8 | | | | | | ✓ | |
| BDBV | Bundibugyo | 5.6 | | | | | | ✓ | |
| SUDV | Boniface | 6.3 | | | | | | ✓ | |
| SUDV | Gulu | 6.5 | | | | | | ✓ | |
| MARV | Ci67 | 8.1 | | | | | | | ✓ |
| MARV | Musoke | 6.5 | | | | | | | ✓ |
| MARV | Angola | 7.6 | | | | | | | ✓ |
| RAVV | Ravn | 7.3 | | | | | | | ✓ |
| WNV | NY99 | 8.5 | | | | | | | |
| WNV | EG101 | 7.1 | | | | | | | |
| CHIKV | B8635 | 7.9 | | | | | | | |
| CHIKV | INDO2354 | 8.3 | | | | | | | |
| JEV | TVP1876 | 8.4 | | | | | | | |
| ZIKV | MK2 | 6.9 | | | | | | | |
| ZIKV | PRVABC59 | 7.9 | | | | | | | |
| JUNV | Candid | 6.7 | | | | | | | |
| RRV | T48 | 8.6 | | | | | | | |
| SLEV | Parton | 7.4 | | | | | | | |
| ONNV | Gulu | 8.1 | | | | | | | |
| SINV | UGMP6440 | 9.0 | | | | | | | |
| SFKV | Unknown | 8.9 | | | | | | | |
| PTV | Unknown | Unknown | | | | | | | |
| VEEV | TC83 | Unknown | | | | | | | |
| VACV | WR | 7.4 | | | | | | | |
| SARS-CoV-2 | WA1 | 6.7 | | | | | | | |

**Supplemental Table 2.** Comparison of the LOD of the CCHFV, LASV, and OMVs AuNP vs. CNB LFIs to additional virus isolates.

| **Virus** | Isolate | **AuNP LFI ($log_{10}$ PFU/mL)** | **CNB LFI ($log_{10}$ PFU/mL)** |
|---|---|---|---|
| CCHFV | Ibr10200 | 6.8 | 6.1 |
| | Afg09 | 6.2 | 5.5 |
| | Hoti | 5.5 | 5.5 |
| | DAK 8194 | NT | ND* |
| | UG3010 | NT | 5.4 |
| | Drosdov | NT | 5.0 |
| | SPU 128/81/7 | NT | 6.3 |
| | PAK JD206 | NT | 4.5 |
| | Chinese HY-13 | NT | 5.4 |
| LASV | Macenta | 6.5 | 5.5 |
| | Pinneo | ND | ND |
| | Josiah | 2.7 | 3.4 |
| | Weller | 2.5 | 3.5 |
| OMVs | Angola | 5.2 | 3.5 |
| | Ci67 | 5.8 | 3.8 |
| | Musoke | 4.7 | 4.0 |
| | Ravn | 4.6 | 3.3 |

*Likely not detected due to initial low titer virus stock tested at 4.9 $log_{10}$ PFU/mL

**Supplemental Table 3.** The LODs of the individual AuNP LFIs in multiple matrices. Recombinant protein or inactivated virus stocks were used for matrices testing.

| Virus | Serum LOD | Soil, tap water, sewage, dust, and mosquito homogenate LOD |
|---|---|---|
| DENV-1 | 62.6 ng/mL | 31.3 ng/mL |
| DENV-2 | 31.2 ng/mL | 15.6 ng/mL |
| DENV-3 | 62.6 ng/mL | 31.3 ng/mL |
| DENV-4 | 31.2 ng/mL | 15.6 ng/mL |
| YFV | 6.1 $\log_{10}$ PFU/mL | 6.1 $\log_{10}$ PFU/mL |
| RVFV | 6.9 $\log_{10}$ PFU/mL | 6.6 $\log_{10}$ PFU/mL |
| CCHFV (Afg09) | 2.5 ng/mL | 2.5 ng/mL |
| LASV (Josiah) | 3.9 ng/mL | 3.9 ng/mL |
| RAVV (Ravn) | 28.5 ng/mL | 28.5 ng/mL |
| EBOV | 6.5 $\log_{10}$ PFU/mL | 6.5 $\log_{10}$ PFU/mL |
